# Supplementary material for: Mechanisms of electrochemical hydrogenation of aromatic compound mixtures over a bimetallic PtRu catalyst
Source: Commun Chem. 2025 Feb 23;8:56. doi: 10.1038/s42004-025-01413-5 (PMC11847916; doi:10.1038/s42004-025-01413-5)

Mechanisms of electrochemical hydrogenation of aromatic compound mixtures over a bimetallic PtRu catalyst

Authors: Cesar Catizane[1], Ying Jiang[1,2]* and Joy Sumner[1*]

1. School of Water, Energy and Environment, Cranfield University, Cranfield, MK43 0AL, UK

2. Renewable and Sustainable Energy Research Centre, Technology Innovation Institute, Abu Dhabi, UAE

*Corresponding authors: ying.jiang@tii.ae, j.sumner@cranfield.ac.uk

Figure S1 – Relative energies of one-to-one hydrogenation step of Benzoic acid into Cyclohexane carboxylic acid. Grey spheres represent carbons, white spheres hydrogens and red oxygens. The optimal condition for each step is highlighted in blue and placed on the lower line. All energies are calculated in difference to the optimal state

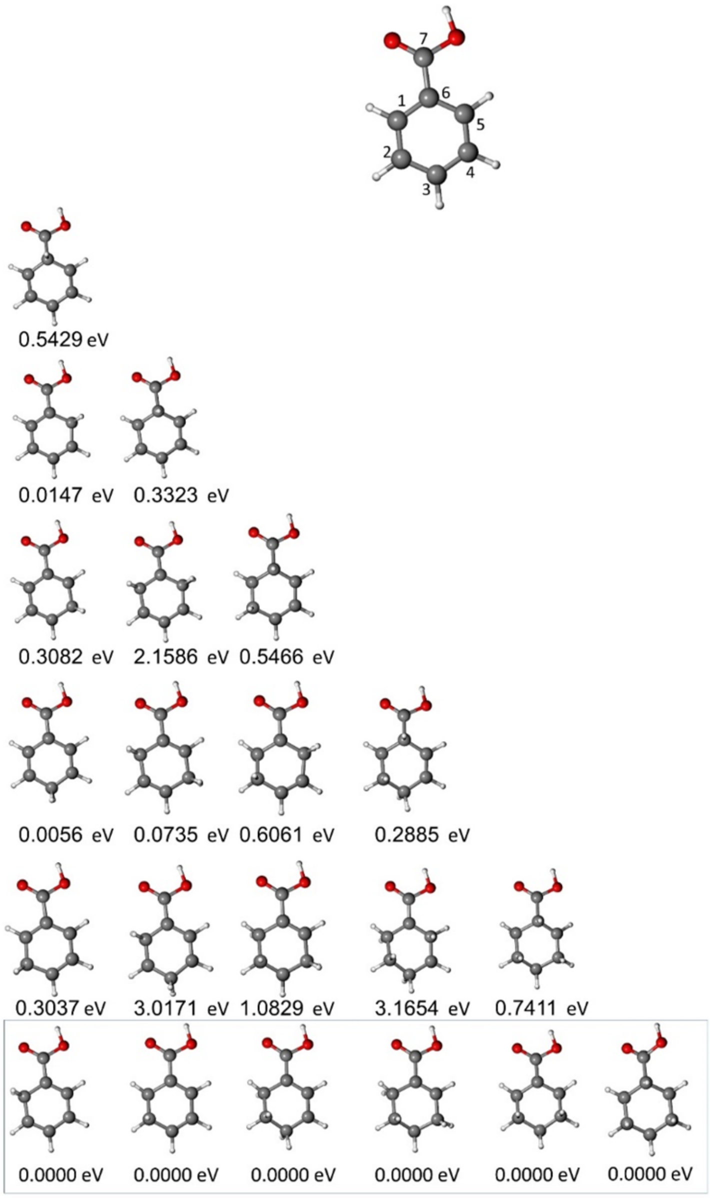

LSV analysis

Figure S2 – Zoomed in Linear-sweep voltammetry of PtRu/ACC before (in yellow) and after the addition BA (in black), P (in red) and BA+P (in light blue), into the system (1.0M H_2SO_4, 55°C). Where ΔE_2, ΔE_3 and ΔE_4 are the differences between no added compound and BA, BA+P and P at 10 mA cm^{-2} respectively. (BA = benzoic acid, P = phenol)

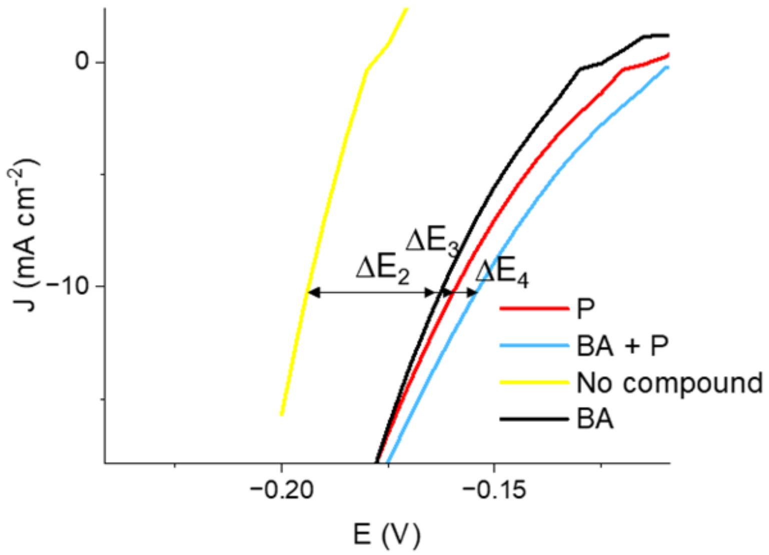

Figure S3 – Zoomed in Linear-sweep voltammetry of PtRu/ACC before (in yellow) and after the addition of G (in green), G+P (in orange) and P (in red) into the system (1.0M H_2SO_4, 55°C). Where ΔE_1, ΔE_3 and ΔE_5 are the differences between no added compound and G, P and G+P at 10 mA cm^{-2} respectively. (G = guaiacol, P = phenol)

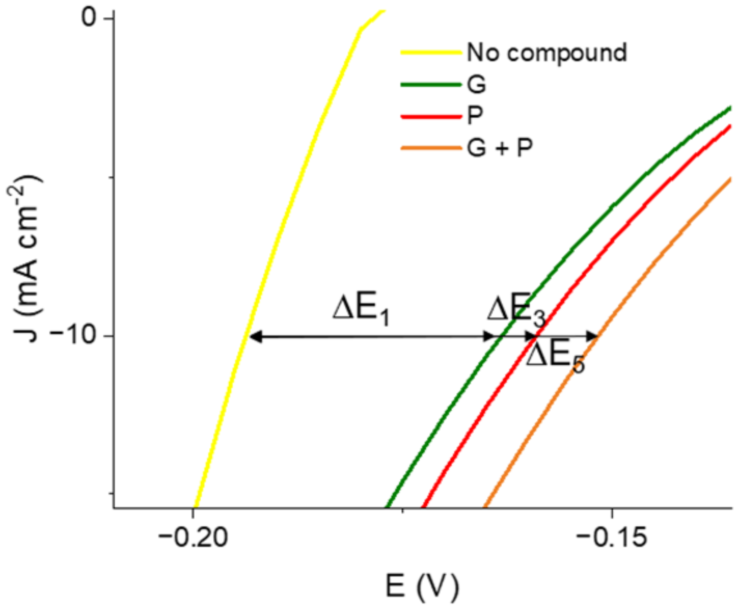

Figure S4 – Zoomed in Linear-sweep voltammetry of PtRu/ACC before (in yellow) and after the addition of BA (in black), BA+G (in pink) and G (in green) into the system (1.0M H_2SO_4, 55°C). Where ΔE_1, ΔE_6 and ΔE_2 are the differences between no added compound and G, BA+G and BA at 10 mA cm^{-2} respectively. (BA = benzoic acid, G = guaiacol)

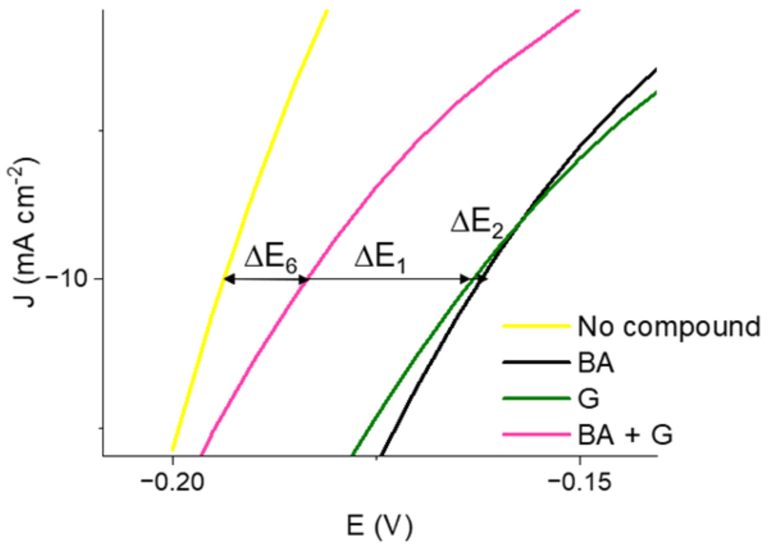

Figure S5 – Zoomed in Linear-sweep voltammetry of PtRu/ACC before (in yellow) and after the addition of BA (in black), G (in green), P (in red) and BA+P+G (in purple) into the system (1.0M H_2SO_4, 55°C). Where ΔE_1, ΔE_2, ΔE_3 and ΔE_7 are the differences between no added compound and G, BA, P and BA+P+G at 10 mA cm^{-2} respectively.

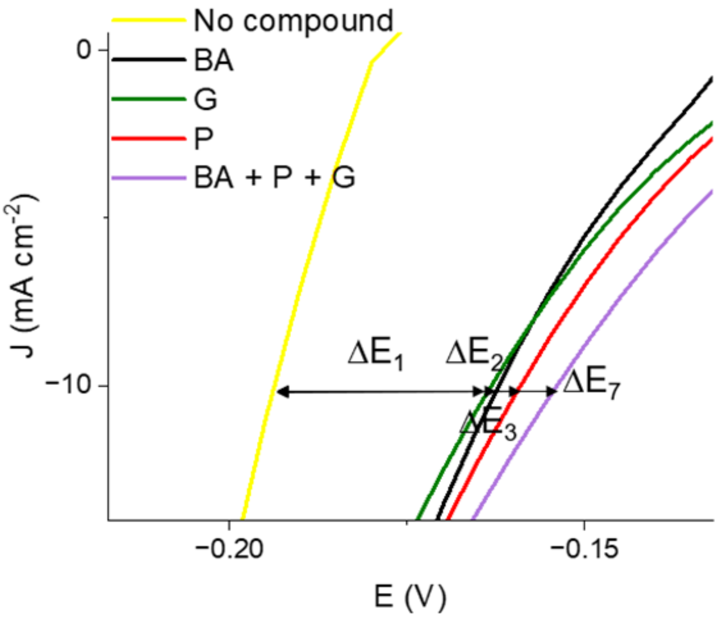

GC-MS spectrum

Figure S6 – GC-MS spectrum of benzoic acid before ECH.

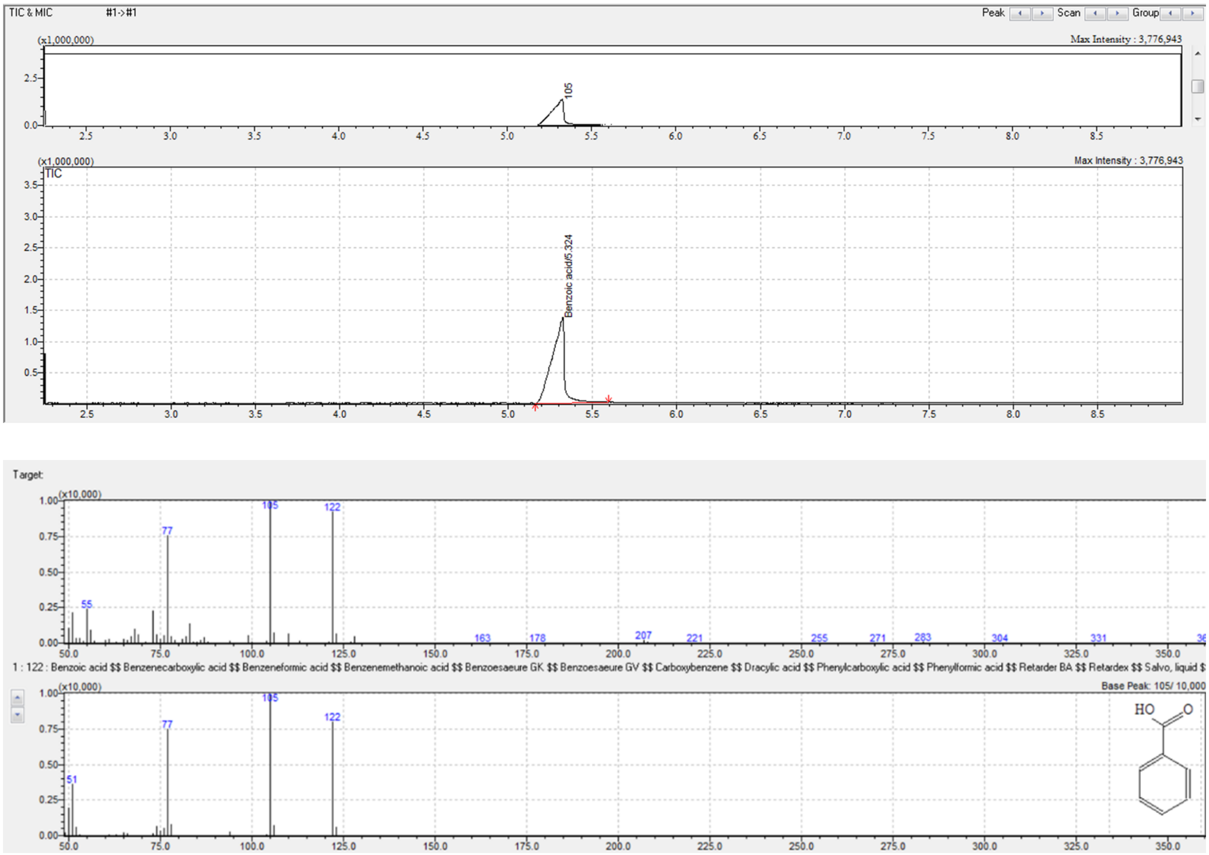

Figure S7 – GC-MS spectrum of benzoic acid after ECH (a) and cyclohexane carboxylic acid (b).

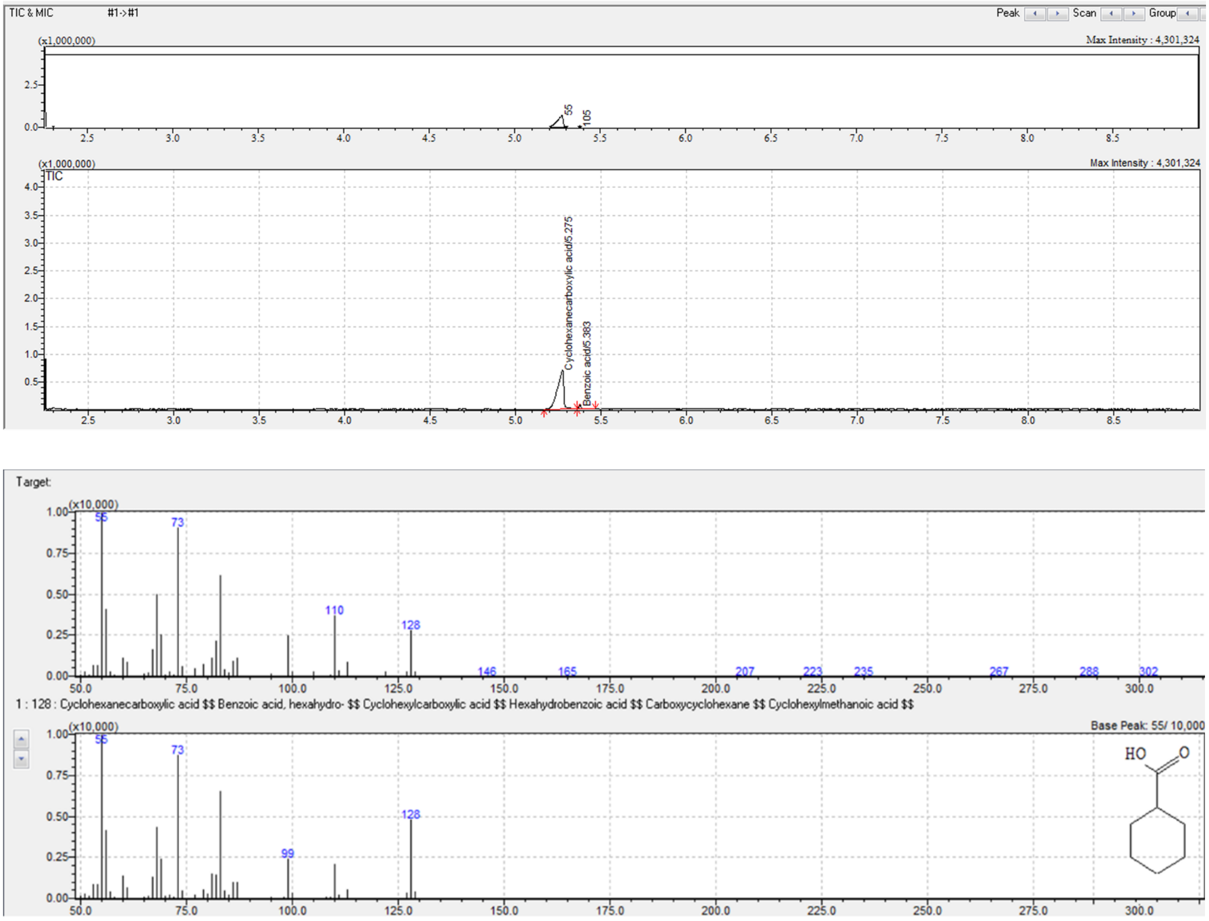

Figure S8 – GC-MS spectrum of phenol before ECH.

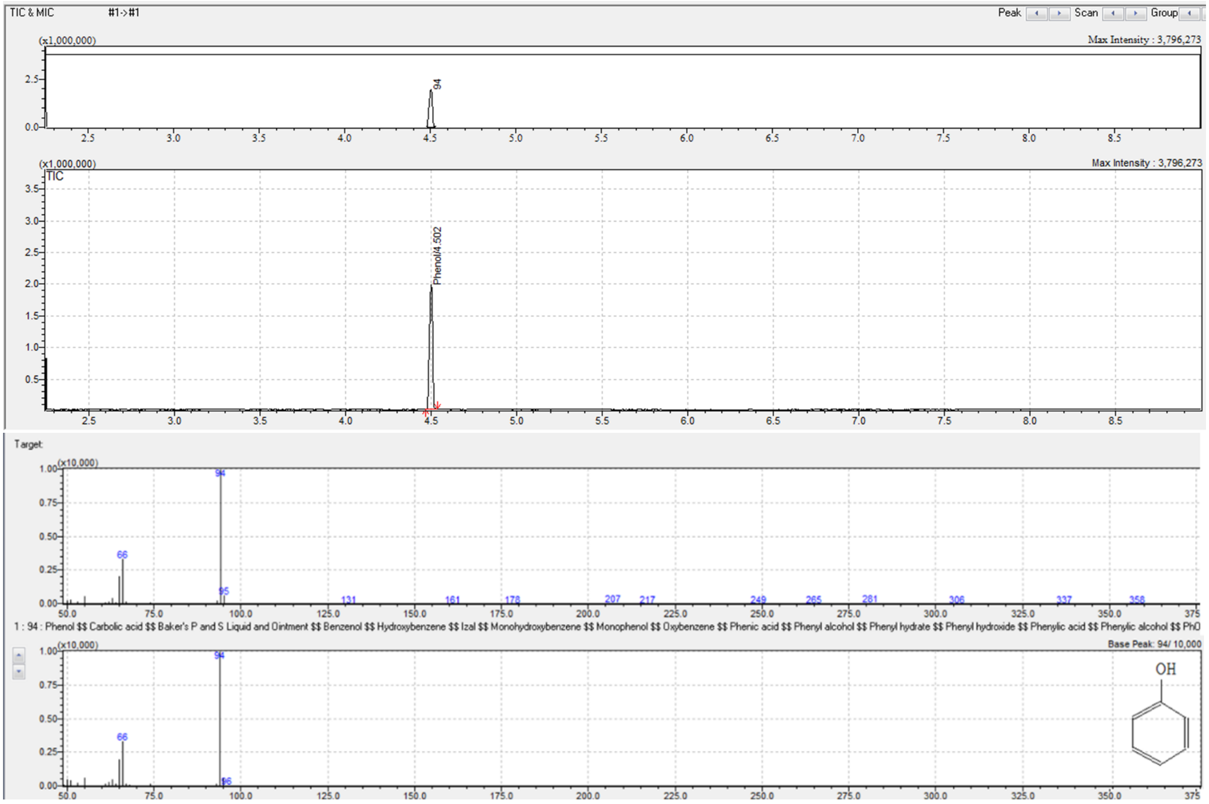

Figure S9 – GC-MS spectrum of phenol after ECH (a), cyclohexanol (b) and cyclohexanone (c).

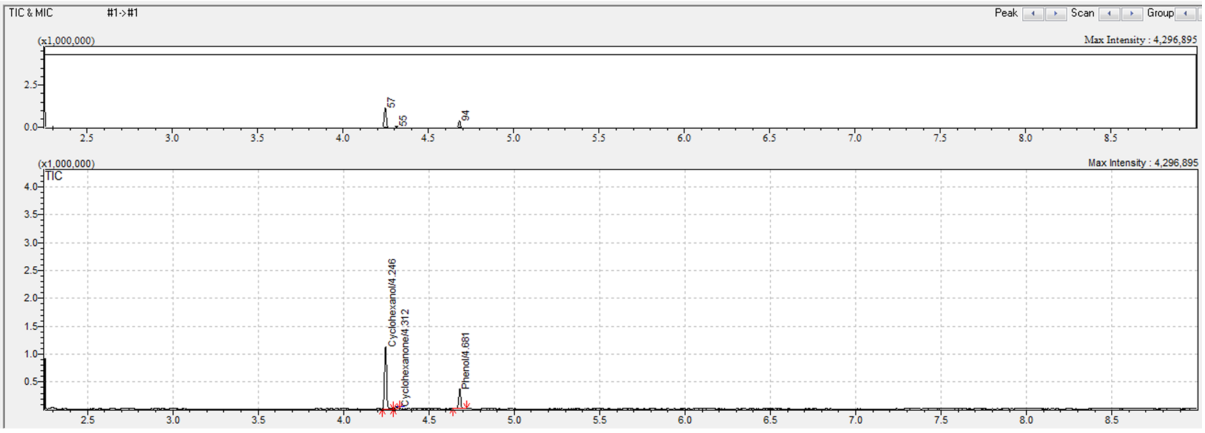

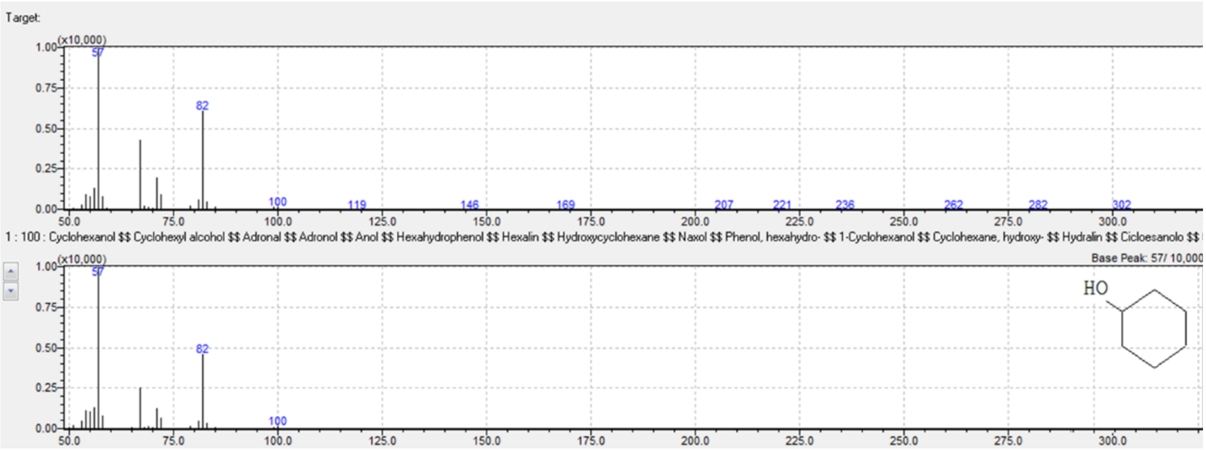

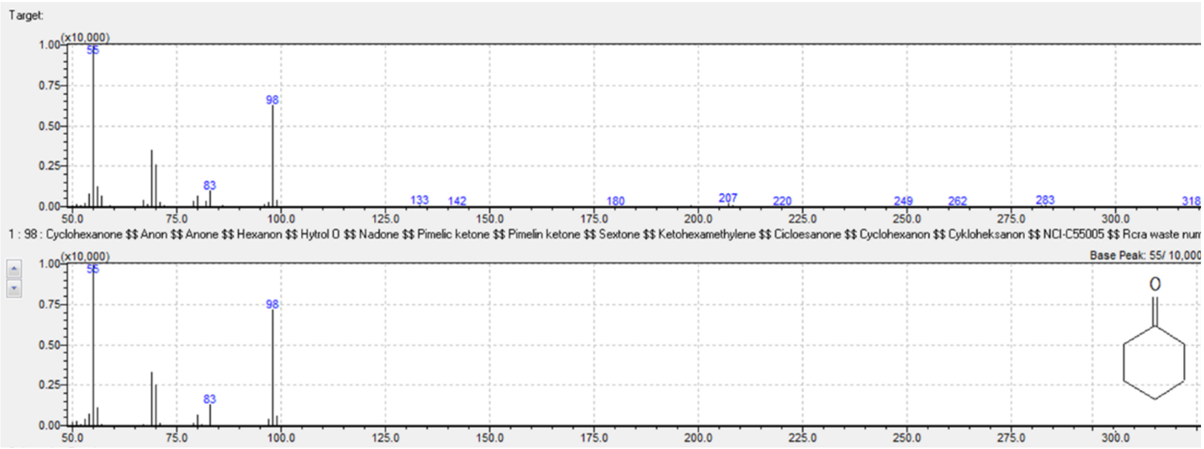

Figure S10 – GC-MS spectrum of guaiacol before ECH.

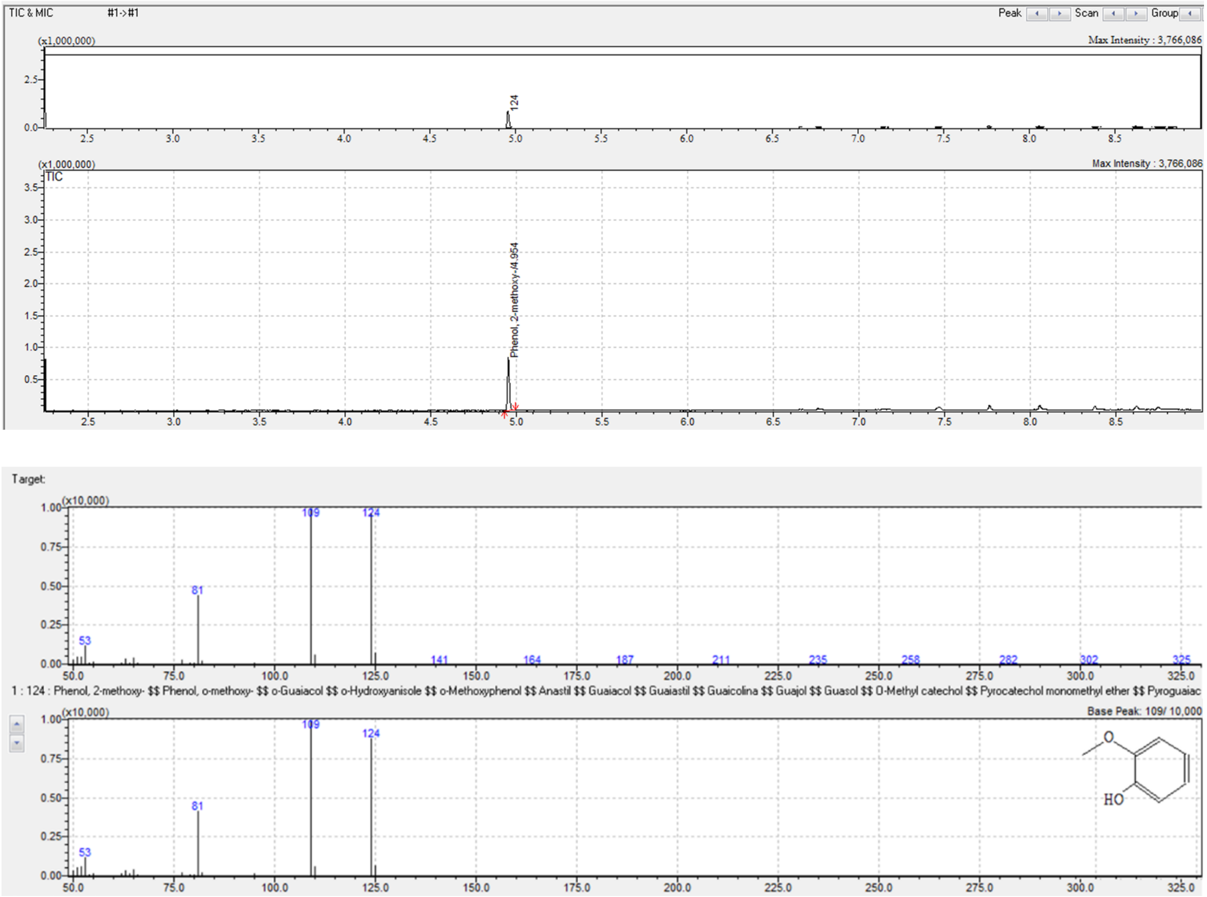

Figure S11 – GC-MS spectrum of guaiacol after ECH (a), methoxycyclohexanol (b) and methoxyclyclohexane (c).

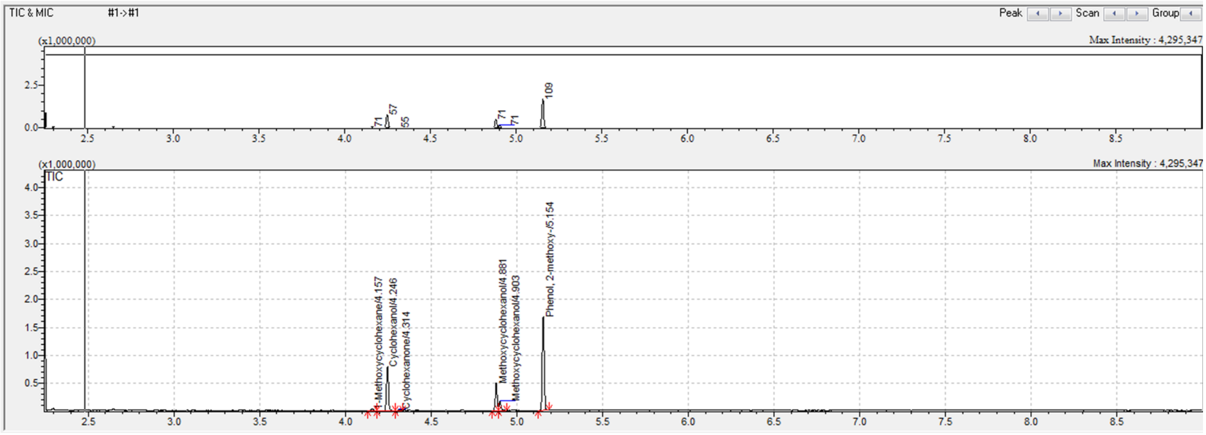

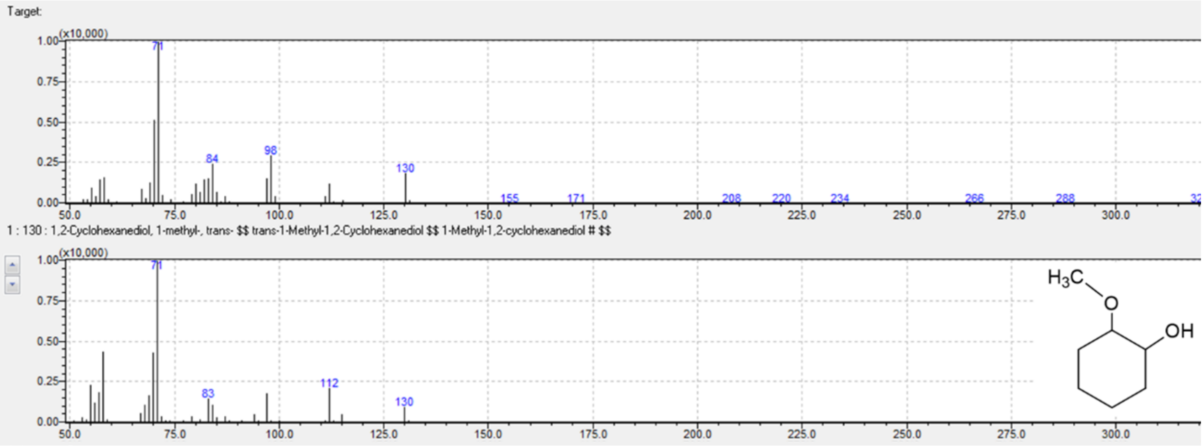

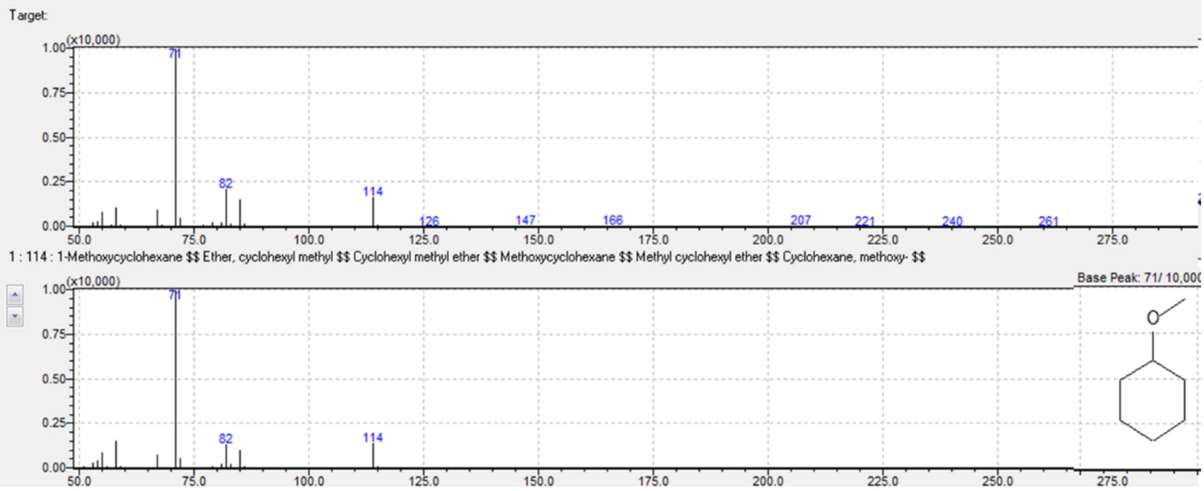

Figure S12 – GC-MS spectrum of benzoic acid and guaiacol mixture before (a) and after (b) ECH. (c) Spectrum of cyclohexane.

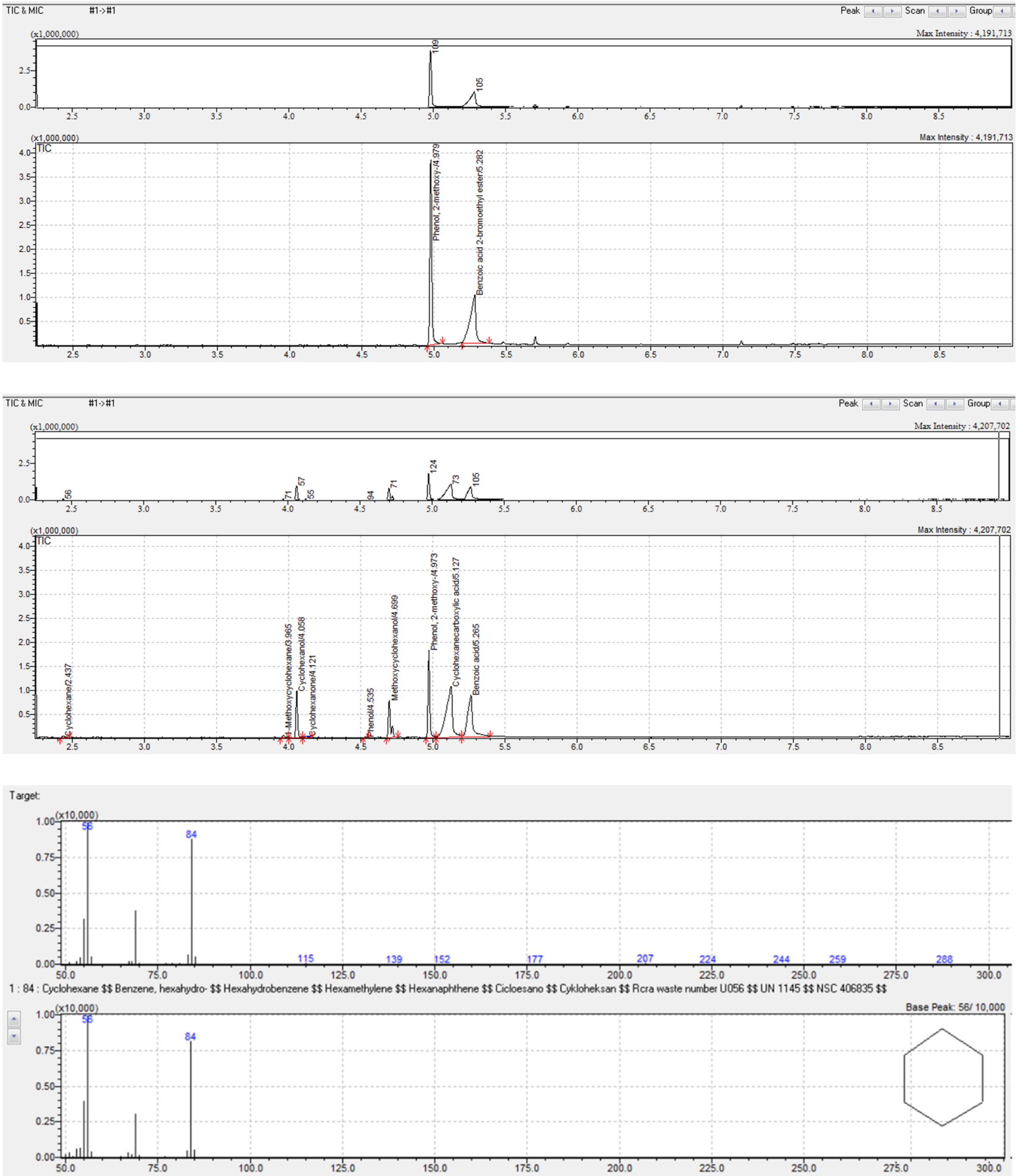

Figure S13 – GC-MS spectrum of benzoic acid and phenol mixture before (a) and after (b) ECH.

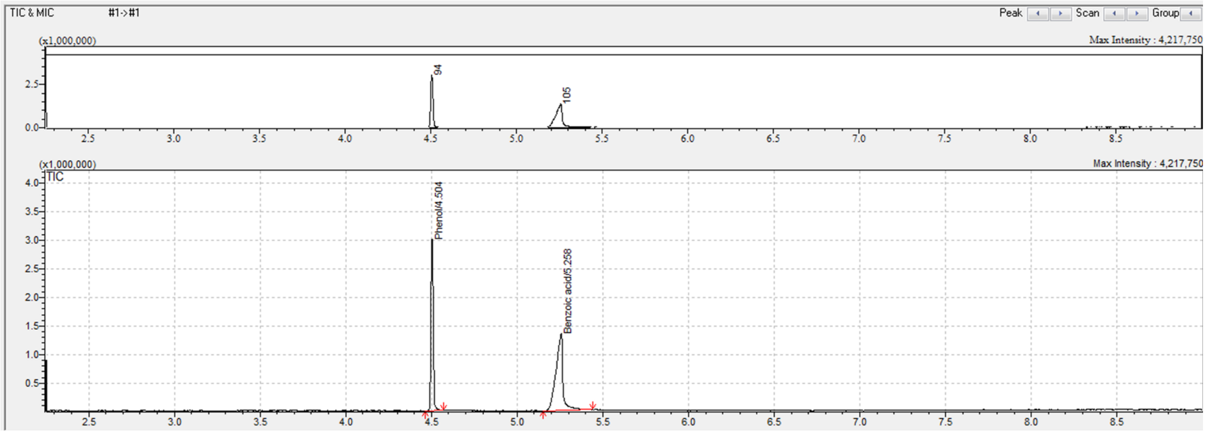

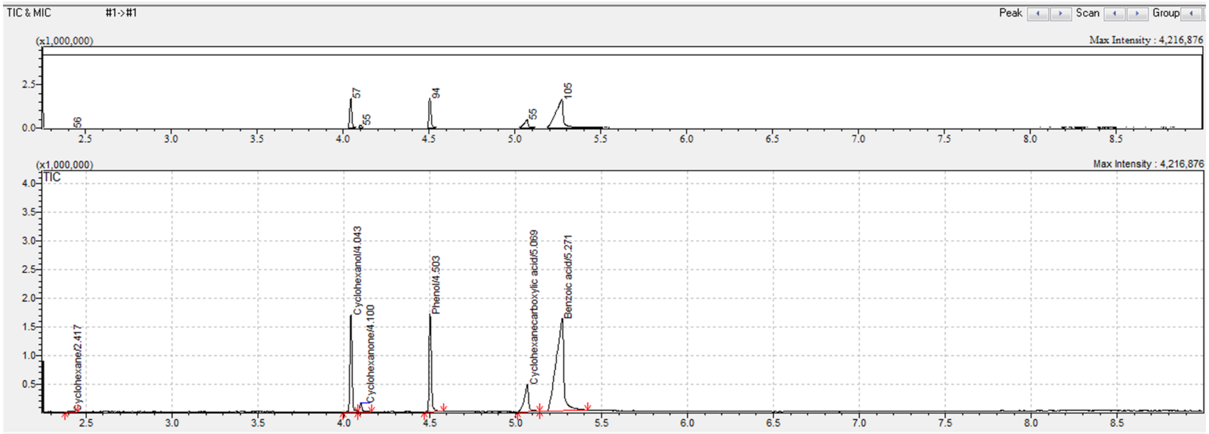

Figure S14 – GC-MS spectrum of guaiacol and phenol mixture before (a) and after (b) ECH.

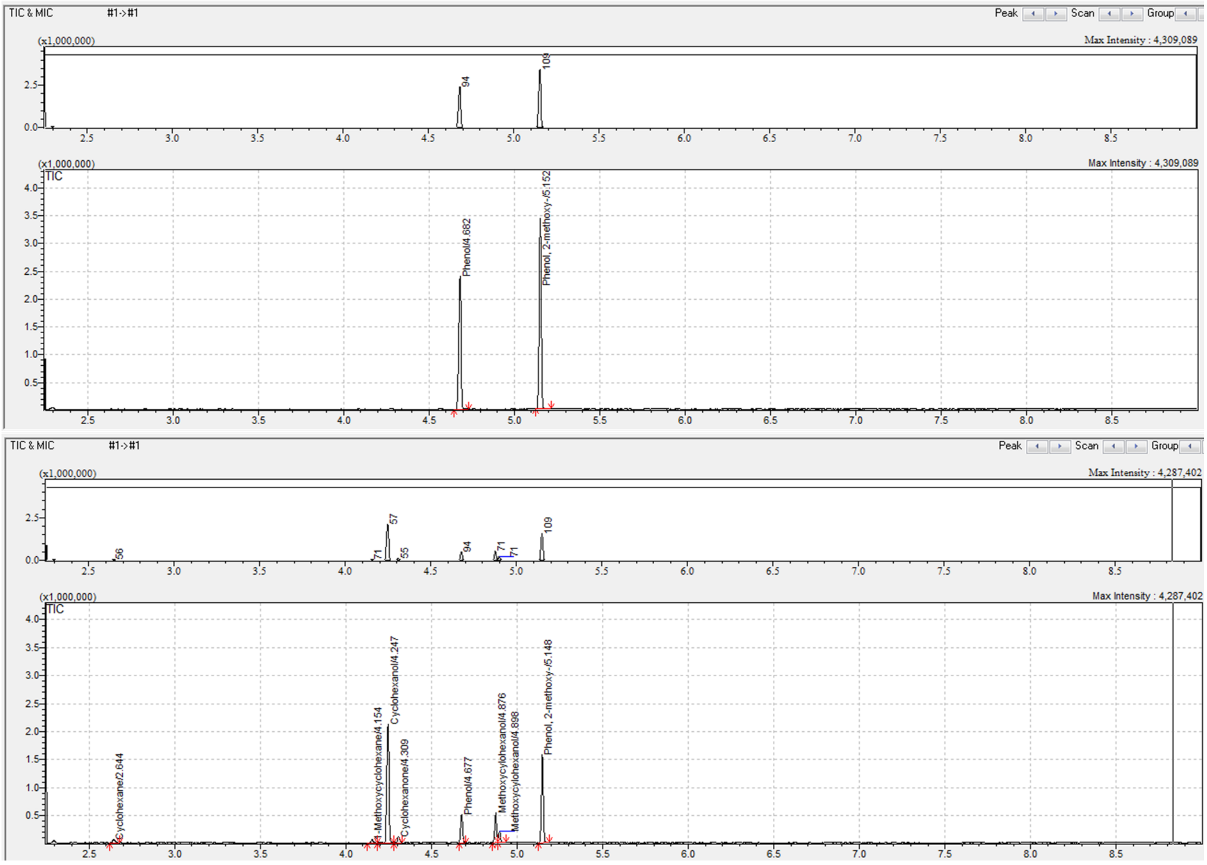

Figure S15 – GC-MS spectrum of benzoic acid, guaiacol and phenol mixture before (a) and after (b) ECH.

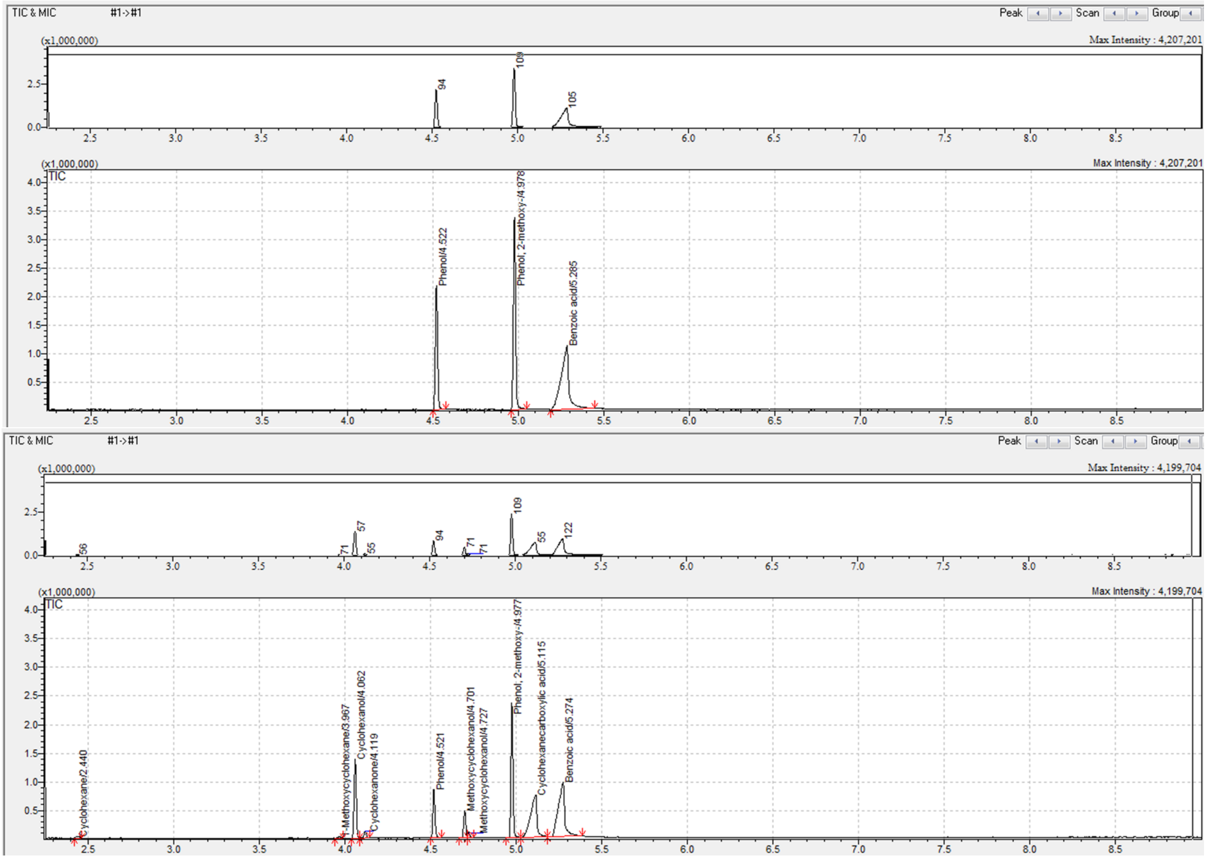

Supplement: Supplementary file 2 — Supplementary Information [file 42004_2025_1413_MOESM2_ESM.pdf]
