# Supplementary material for: Mechanisms of electrochemical hydrogenation of aromatic compound mixtures over a bimetallic PtRu catalyst
Source: Commun Chem. 2025 Feb 23;8:56. doi: 10.1038/s42004-025-01413-5 (PMC11847916; doi:10.1038/s42004-025-01413-5)
Supplement: Supplementary file 3 — Description of Additional Supplementary Files [file 42004_2025_1413_MOESM3_ESM.pdf]

Description of Additional Supplementary Files

File name- Supplementary Data 1

File description- Atom positions after the optimisation of each system.
