# Supplementary material for: Mechanisms of electrochemical hydrogenation of aromatic compound mixtures over a bimetallic PtRu catalyst
Source: Commun Chem. 2025 Feb 23;8:56. doi: 10.1038/s42004-025-01413-5 (PMC11847916; doi:10.1038/s42004-025-01413-5)
Supplement: Supplementary file 4 — Supplementary Data 1 [file 42004_2025_1413_MOESM4_ESM.docx]

Figure 7a - Slab + benzoic acid (vertical)

Ru -0.075667 3.021157 8.334266

Pt 3.896718 0.717619 8.440524

Pt 2.568648 3.016603 8.442644

Ru 1.254120 0.724350 8.334577

Ru 2.528302 1.461483 6.192847

Pt -0.131365 1.473089 6.098013

Pt -1.463229 3.782126 6.097213

Ru 1.194635 3.774431 6.191976

Ru 2.668561 4.622082 3.837360 0 0 0

Pt 1.315279 2.336408 3.796152 0 0 0

Pt 0.012468 4.592944 3.796152 0 0 0

Ru -1.302812 2.329209 3.785974 0 0 0

Ru -0.000000 3.081388 1.658490 0 0 0

Pt 3.983840 0.795714 1.617282 0 0 0

Pt 2.681028 3.052250 1.617282 0 0 0

Ru 1.365748 0.788515 1.607103 0 0 0

Ru -2.760178 7.668978 8.332606

Pt 1.227391 5.322234 8.429069

Pt -0.120802 7.648457 8.437059

Ru -1.414266 5.345730 8.326584

Ru -0.134559 6.086316 6.186943

Pt -2.793694 6.088674 6.097352

Pt -4.124993 8.398104 6.096573

Ru -1.469049 8.393254 6.190046

Ru 5.337121 0.000000 3.837360 0 0 0

Pt -1.353281 6.958490 3.796152 0 0 0

Pt -2.656093 9.215026 3.796152 0 0 0

Ru -3.971373 6.951292 3.785974 0 0 0

Ru -2.668561 7.703471 1.658490 0 0 0

Pt 1.315279 5.417796 1.617282 0 0 0

Pt 0.012468 7.674332 1.617282 0 0 0

Ru -1.302812 5.410598 1.607103 0 0 0

Ru 5.262764 3.043428 8.321755

Pt 9.243093 0.717734 8.453010

Pt 7.907451 3.024986 8.433746

Ru 6.584673 0.736360 8.361407

Ru 7.865882 1.465343 6.173006

Pt 5.207962 1.463566 6.104985

Pt 3.869556 3.781323 6.102968

Ru 6.529527 3.771619 6.181234

Ru -2.668561 4.622082 3.837360 0 0 0

Pt 6.652401 2.336408 3.796152 0 0 0

Pt 5.349589 4.592944 3.796152 0 0 0

Ru 4.034309 2.329209 3.785974 0 0 0

Ru 5.337121 3.081388 1.658490 0 0 0

Pt 9.320961 0.795714 1.617282 0 0 0

Pt 8.018149 3.052250 1.617282 0 0 0

Ru 6.702869 0.788515 1.607103 0 0 0

Ru 2.553160 7.656953 8.517754

Pt 6.575898 5.332742 8.434028

Pt 5.228309 7.634985 8.434470

Ru 3.938027 5.317409 8.357555

Ru 5.201861 6.084232 6.179652

Pt 2.549172 6.101717 6.122596

Pt 1.221859 8.400069 6.121057

Ru 3.883498 8.405325 6.146037

Ru 0.000000 0.000000 3.837360 0 0 0

Pt 3.983840 6.958490 3.796152 0 0 0

Pt 2.681028 9.215026 3.796152 0 0 0

Ru 1.365748 6.951292 3.785974 0 0 0

Ru 2.668561 7.703471 1.658490 0 0 0

Pt 6.652401 5.417796 1.617282 0 0 0

Pt 5.349589 7.674332 1.617282 0 0 0

Ru 4.034309 5.410598 1.607103 0 0 0

C 1.927537 6.306026 13.134089

C 1.724709 5.713992 14.381731

C 2.460150 6.152097 15.493594

C 3.397065 7.187950 15.358728

C 3.594943 7.796438 14.116066

C 2.861570 7.353136 12.998235

H 4.324623 8.610558 13.998937

H 3.984108 7.517191 16.229297

H 2.302672 5.679255 16.475133

H 0.992912 4.898423 14.485154

H 1.368264 5.971638 12.247826

C 3.069926 7.941814 11.665211

O 3.915400 8.970275 11.592187

O 2.460403 7.452069 10.662880

H 3.959901 9.285543 10.607803

Figure 7b - Slab + benzoic acid (horizontal)

Ru -0.074714 3.027749 8.348143

Pt 3.901980 0.685917 8.406679

Pt 2.598986 3.035939 8.557825

Ru 1.252862 0.733644 8.339212

Ru 2.600926 1.520953 6.150171

Pt -0.063652 1.506156 6.088809

Pt -1.393298 3.814627 6.084155

Ru 1.282270 3.805357 6.154131

Ru 2.668561 4.622082 3.837360 0 0 0

Pt 1.315279 2.336408 3.796152 0 0 0

Pt 0.012468 4.592944 3.796152 0 0 0

Ru -1.302812 2.329209 3.785974 0 0 0

Ru 0.000000 3.081388 1.658490 0 0 0

Pt 3.983840 0.795714 1.617282 0 0 0

Pt 2.681028 3.052250 1.617282 0 0 0

Ru 1.365748 0.788515 1.607103 0 0 0

Ru -2.754179 7.661480 8.369491

Pt 1.239133 5.338202 8.401650

Pt -0.100785 7.640449 8.434941

Ru -1.402954 5.332499 8.441828

Ru -0.059117 6.128539 6.129604

Pt -2.745342 6.122944 6.137829

Pt -4.059519 8.435517 6.083177

Ru -1.396169 8.435053 6.138046

Ru 5.337121 0.000000 3.837360 0 0 0

Pt -1.353281 6.958490 3.796152 0 0 0

Pt -2.656093 9.215026 3.796152 0 0 0

Ru -3.971373 6.951292 3.785974 0 0 0

Ru -2.668561 7.703471 1.658490 0 0 0

Pt 1.315279 5.417796 1.617282 0 0 0

Pt 0.012468 7.674332 1.617282 0 0 0

Ru -1.302812 5.410598 1.607103 0 0 0

Ru 5.300940 2.933952 8.662556

Pt 9.258224 0.725775 8.418871

Pt 7.941037 3.021158 8.414008

Ru 6.609699 0.721728 8.356284

Ru 7.929153 1.505401 6.128652

Pt 5.269333 1.514924 6.112330

Pt 3.950918 3.821743 6.172842

Ru 6.611173 3.832919 6.124612

Ru -2.666651 4.622082 3.837360 0 0 0

Pt 6.652401 2.336408 3.796152 0 0 0

Pt 5.349589 4.592944 3.796152 0 0 0

Ru 4.034309 2.329209 3.785974 0 0 0

Ru 5.337121 3.081388 1.658490 0 0 0

Pt 9.320961 0.795714 1.617282 0 0 0

Pt 8.018149 3.052250 1.617282 0 0 0

Ru 6.702869 0.788515 1.607103 0 0 0

Ru 2.586143 7.680851 8.382978

Pt 6.568487 5.338790 8.578031

Pt 5.251911 7.678694 8.407442

Ru 3.850120 5.436034 8.683391

Ru 5.291784 6.123872 6.115476

Pt 2.613106 6.119367 6.124443

Pt 1.271658 8.434942 6.110933

Ru 3.938160 8.427922 6.133348

Ru 0.000000 0.000000 3.837360 0 0 0

Pt 3.983840 6.958490 3.796152 0 0 0

Pt 2.681028 9.215026 3.796152 0 0 0

Ru 1.365748 6.951292 3.785974 0 0 0

Ru 2.668561 7.703471 1.658490 0 0 0

Pt 6.652401 5.417796 1.617282 0 0 0

Pt 5.349589 7.674332 1.617282 0 0 0

Ru 4.034309 5.410598 1.607103 0 0 0

C 3.288316 4.857787 10.679035

C 3.262463 3.392904 10.628889

C 4.573977 2.737471 10.706783

C 5.795536 3.479498 10.700242

C 5.833058 4.947411 10.629448

C 4.518159 5.614605 10.730985

H 6.650882 5.429867 11.174792

H 6.703974 2.985072 11.047465

H 4.595009 1.709639 11.072073

H 2.448040 2.909246 11.179486

H 2.395996 5.364261 11.050242

C 4.392610 6.973158 11.340542

O 5.562349 7.431483 11.873241

O 3.344836 7.604338 11.435526

H 5.365708 8.327025 12.219188

Figure 7c - Slab + benzoic acid (horizontal)

Ru -0.104489 3.029772 8.606660

Pt 3.922791 0.707133 8.387160

Pt 2.632503 3.006530 8.527789

Ru 1.267806 0.692624 8.336488

Ru 2.561031 1.488364 6.145879

Pt -0.090066 1.490030 6.111667

Pt -1.421369 3.800729 6.101313

Ru 1.243177 3.790114 6.127136

Ru 2.668561 4.622082 3.837360 0 0 0

Pt 1.315279 2.336408 3.796152 0 0 0

Pt 0.012468 4.592944 3.796152 0 0 0

Ru -1.302812 2.329209 3.785974 0 0 0

Ru -0.000000 3.081388 1.658490 0 0 0

Pt 3.983840 0.795714 1.617282 0 0 0

Pt 2.681028 3.052250 1.617282 0 0 0

Ru 1.365748 0.788515 1.607103 0 0 0

Ru -2.736251 7.668732 8.308476

Pt 1.248405 5.394724 8.454509

Pt -0.106655 7.673134 8.384507

Ru -1.440231 5.376854 8.332830

Ru -0.095717 6.099350 6.127899

Pt -2.758815 6.097373 6.092217

Pt -4.105721 8.428776 6.092071

Ru -1.435913 8.415767 6.139550

Ru 5.337121 0.000000 3.837360 0 0 0

Pt -1.353281 6.958490 3.796152 0 0 0

Pt -2.656093 9.215026 3.796152 0 0 0

Ru -3.971373 6.951292 3.785974 0 0 0

Ru -2.668561 7.703471 1.658490 0 0 0

Pt 1.315279 5.417796 1.617282 0 0 0

Pt 0.012468 7.674332 1.617282 0 0 0

Ru -1.302812 5.410598 1.607103 0 0 0

Ru 5.301002 3.032992 8.320951

Pt 9.251114 0.726185 8.415793

Pt 7.919774 3.037601 8.403263

Ru 6.595955 0.740195 8.348247

Ru 7.914952 1.485334 6.146633

Pt 5.249980 1.484310 6.085217

Pt 3.898319 3.785423 6.119089

Ru 6.570185 3.794836 6.155971

Ru -2.668561 4.622082 3.837360 0 0 0

Pt 6.652401 2.336408 3.796152 0 0 0

Pt 5.349589 4.592944 3.796152 0 0 0

Ru 4.034309 2.329209 3.785974 0 0 0

Ru 5.337121 3.081388 1.658490 0 0 0

Pt 9.320961 0.795714 1.617282 0 0 0

Pt 8.018149 3.052250 1.617282 0 0 0

Ru 6.702869 0.788515 1.607103 0 0 0

Ru 2.597262 7.696680 8.429427

Pt 6.584602 5.347552 8.409476

Pt 5.253575 7.648640 8.408277

Ru 3.970846 5.359804 8.365149

Ru 5.222447 6.096417 6.181783

Pt 2.562871 6.105379 6.121945

Pt 1.249989 8.422586 6.099401

Ru 3.899859 8.417303 6.174534

Ru 0.000000 0.000000 3.837360 0 0 0

Pt 3.983840 6.958490 3.796152 0 0 0

Pt 2.681028 9.215026 3.796152 0 0 0

Ru 1.365748 6.951292 3.785974 0 0 0

Ru 2.668561 7.703471 1.658490 0 0 0

Pt 6.652401 5.417796 1.617282 0 0 0

Pt 5.349589 7.674332 1.617282 0 0 0

Ru 4.034309 5.410598 1.607103 0 0 0

C 0.810730 5.178698 10.548027

C -0.021987 3.958716 10.627419

C 0.619056 2.683749 10.659036

C 2.093445 2.643337 10.657857

C 2.835738 3.828603 10.990972

C 2.216221 5.087232 10.964592

H 3.887882 3.743841 11.277381

H 2.552725 1.685488 10.927510

H 0.093627 1.823788 11.088047

H -1.031576 4.065374 11.039039

H 0.303865 6.102307 10.855065

C 2.946553 6.322600 11.192846

O 4.040216 6.209260 11.987458

O 2.624018 7.438554 10.713267

H 4.521817 7.068089 11.901048
